# Public Awareness and Application of the Heimlich Maneuver: A Survey of Shenzhen Residents

**DOI:** 10.7759/cureus.88957

**Published:** 2025-07-29

**Authors:** Jiao Yang, Zhijuan Ling, Yong-chao Chen, Xin Wang, Hongguang Pan

**Affiliations:** 1 Department of Otorhinolaryngology, Shenzhen Children's Hospital, Shenzhen, CHN

**Keywords:** airway obstruction, awareness, emergency training, heimlich maneuver, public health

## Abstract

Purpose: This study evaluates the awareness and practical application of the Heimlich Maneuver among residents of Shenzhen, a rapidly urbanizing region in China, to address the gap in region-specific first aid knowledge. It examines factors influencing awareness and application and explores the relationship between training, knowledge, and behavior to inform public health interventions.

Methods: A cross-sectional survey was conducted among Shenzhen residents aged 18 years and older using a self-administered questionnaire. The questionnaire covered demographics, awareness, knowledge, application, and learning behaviors. Data were analyzed using SPSS software, version 26.0 (IBM Corp., Armonk, NY), employing Chi-square tests and correlation analyses to assess associations between demographic factors and awareness.

Results: Of 492 valid responses, 94.1% were aware of the Heimlich Maneuver, but only 40.9% knew how to perform it. Awareness was significantly associated with gender (p = 0.013), age (p = 0.005), occupation (p = 0.014), and education (p = 0.037). Healthcare professionals and those with higher education demonstrated greater knowledge. Only 27.6% had received training, with 34.6% of trained individuals applying the maneuver in real-life scenarios. Awareness correlated positively with training (χ² = 24.003, p < 0.01) and application (χ² = 7.868, p = 0.049). Most respondents (81.9%) were willing to learn, preferring short videos and community activities.

Conclusion: Despite high awareness, the practical application of the Heimlich Maneuver in Shenzhen remains limited. Targeted, hands-on training using modern media and community programs can enhance skills, particularly among healthcare workers and educated individuals, supporting effective public health education strategies.

## Introduction

Airway obstruction due to foreign bodies is a critical emergency in otolaryngology, particularly affecting children under five years and, increasingly, elderly individuals due to improper dietary habits [1-3]. This demographic shift highlights the rising prevalence and complexity of airway foreign body incidents, necessitating effective emergency interventions to reduce mortality [4]. The Heimlich Maneuver, a simple yet effective technique for dislodging airway obstructions, is widely recognized for its life-saving potential [5]. Despite its accessibility, public awareness and proficiency in this technique remain limited, particularly in urban settings.

Global research on Heimlich Maneuver awareness is sparse, with studies often focusing on specific groups, such as teachers in Saudi Arabia or kindergarten staff in Ethiopia, rather than general urban populations [6-8]. In China, data on public awareness are particularly scarce, especially in rapidly urbanizing cities like Shenzhen, where high population turnover and diverse demographics present unique challenges for health education. Shenzhen’s advanced healthcare infrastructure, combined with its highly transient population, makes it an ideal setting to explore emergency response knowledge gaps. The frequent mobility of residents, many of whom are migrant workers or short-term dwellers, poses significant challenges for consistent public health outreach and continuity of training. This mobility may lead to disparities in access to first aid education, reduced participation in community health programs, and lower retention of practical emergency skills, such as the Heimlich Maneuver.

This study aims to assess the level of awareness and practical application of the Heimlich Maneuver among community residents in Shenzhen. It also seeks to explore the factors influencing awareness and usage, while examining the relationship between training experience, knowledge, and behavior. By surveying the first aid knowledge and behaviors of Shenzhen residents, this research intends to provide data-driven support for the dissemination of first aid knowledge and the development of related training programs, ultimately contributing to the enhancement and optimization of public health education.

## Materials and methods

Study participants

This cross-sectional study was conducted among community residents in Shenzhen, China, to evaluate their awareness and practical application of the Heimlich Maneuver. Participants were selected using a stratified convenience sampling method to ensure representation across different age groups, genders, occupations, and educational levels.

Residents aged 18 years or older who were permanent or long-term community members and able to independently complete the questionnaire were eligible for inclusion. The transient nature of Shenzhen’s population, characterized by a high proportion of migrants and short-term residents, was a key contextual factor, as it influences exposure to consistent health education and may affect emergency response behavior.

Exclusion criteria included: (1) responses that were substantially incomplete (e.g., missing entire sections such as demographics or first aid knowledge), (2) severe internal inconsistencies that compromised data integrity (e.g., reporting complete unawareness of the Heimlich Maneuver but simultaneously claiming frequent use), and (3) self-reported inability to complete the questionnaire due to cognitive or language limitations.

Sample size calculation

The required sample size was estimated using the formula for cross-sectional studies: n = Z² × p × (1-p) / d²; where Z = 1.96 (for 95% confidence level), p = 0.5 (estimated awareness rate), and d = 0.05 (margin of error). This yielded a minimum sample size of 384 participants. Considering a 10% anticipated non-response or invalid rate, the adjusted target sample size was set at 425. A total of 500 questionnaires were distributed, and 492 valid responses were obtained (response rate: 98.4%), exceeding the minimum statistical requirement.

Questionnaire design and distribution

The questionnaire was designed based on a comprehensive literature review and refined through expert consultation with three otolaryngologists from Shenzhen Children's Hospital. It consisted of six sections: (1) demographic information, (2) awareness of the Heimlich Maneuver, (3) general first aid knowledge, (4) practical application experience, (5) willingness to learn, and (6) suggestions for training implementation.

To ensure content validity, the draft questionnaire was reviewed by the expert panel for relevance and clarity. A pilot test was conducted with 30 community participants (15 online, 15 offline) to assess readability and item clarity. Based on the feedback, several items were rephrased for improved comprehension. The reliability of the questionnaire was assessed using Cronbach’s alpha, which yielded a value of 0.81, indicating acceptable internal consistency.

Both online and offline distribution channels were utilized to maximize participation. The online version was disseminated via WeChat using Wenjuanxing (https://www.wjx.cn), a widely used online survey platform in China. Offline surveys were conducted at hospitals and community centers by trained staff, who were available to assist with instructions when needed. Before participation, all respondents received a study overview and were informed of their rights, including voluntary participation, anonymity, and data confidentiality. Two researchers independently screened the returned questionnaires, excluding eight that demonstrated severe logical inconsistencies (e.g., indicating no awareness of the Heimlich Maneuver while simultaneously reporting high proficiency in its use), or that were substantially incomplete (e.g., missing entire demographic sections). Responses from participants who reported performing the maneuver without prior formal training were retained, as they may reflect informal or observational learning and were considered valid for inclusion. The full questionnaire is available in Appendix A as a structured and formatted table.

Statistical analysis

All data were analyzed using IBM SPSS Statistics version 26.0 (IBM Corp., Armonk, NY, USA). Descriptive statistics were used to summarize demographic data and responses across the six modules. Chi-square tests were conducted to examine associations between demographic variables (gender, age, occupation, and education) and awareness levels. Cramér’s V was used to interpret effect sizes. Additionally, Pearson correlation analysis was performed to assess relationships between awareness, training history, and practical application. A two-tailed p-value of <0.05 was considered statistically significant for all tests.

## Results

Demographic characteristics

A total of 492 valid questionnaires (156 paper-based, 336 electronic) were analyzed. Table 1 summarizes the demographic characteristics. Of the respondents, 33.5% (165) were male, and 66.5% (327) were female. The majority (63.4%, 312) were aged 31-45 years, followed by 18-30 years (17.7%, 87), 46-60 years (9.8%, 48), and over 60 years (9.1%, 45). Occupationally, 27.4% (135) were private enterprise employees, 15.7% (77) were healthcare workers, 15.4% (76) were teachers, and 41.5% (204) were in other professions. Educationally, 76.6% (377) had tertiary education or higher, including 36.6% (180) with bachelor’s degrees and 6.1% (30) with graduate degrees or higher; 23.4% (115) had high school education or less.

**Table 1:** Demographic characteristics of respondents

Demographic Information	Paper Questionnaire (n)	Electronic Questionnaire (n)	Total (n)
1. Gender (Male)			
Male	59	106	165
Female	97	230	327
2. Age			
18-30 years	20	67	87
31-45 years	118	194	312
46-60 years	17	65	82
60 years and older	1	10	11
3. Occupation			
Healthcare Workers	6	71	77
Teachers	7	29	36
Students	3	18	21
Private Employees	78	57	135
Retired Individuals	6	14	20
Others	56	147	203
4. Education Level			
Primary or Below	4	2	6
Junior High School	14	42	56
High School	27	38	65
College/Associate Degree	50	105	155
Bachelor's Degree	43	137	180
Graduate or Above	18	12	30

Awareness of the Heimlich maneuver

In terms of awareness, 94.1% (463 individuals) of participants had heard of the Heimlich maneuver. The awareness rate was higher among those who completed the electronic questionnaire (96.4%) compared to those completing the paper questionnaire (92.4%), although this difference was not statistically significant. The participants' understanding of the Heimlich maneuver is presented in Table 2. Of the respondents, 40.9% (201 individuals) knew how to perform the maneuver, 44.3% (218 individuals) understood its purpose, while 10.4% (51 individuals) had only heard the name but were unaware of its function, and 4.5% (22 individuals) had no knowledge at all.

**Table 2:** Awareness of the Heimlich maneuver among respondents *Note: χ² tests; p<0.05 significant.

Demographic Information	Know How to Perform	Understand the Purpose	Heard the Name, But Unaware	Completely Unaware	χ²	P-value
1. Gender (Male)						
Male	67	62	25	11	10.704	0.013
Female	134	156	26	11		
2. Age						
18-30 years	12	30	8	1	23.556	0.005
31-45 years	29	140	43	6		
46-60 years	35	130	35	1		
60 years and older	6	12	1	3		
3. Occupation						
Healthcare Workers	49	22	4	2	29.427	0.014
Teachers	15	15	5	1		
Students	9	9	2	1		
Private Employees	43	65	20	7		
Retired Individuals	4	14	1	1		
Others	81	93	19	10		
4. Education Level						
Primary or Below	0	6	0	0	26.100	0.037
Junior High School	17	24	8	7		
High School	25	34	3	3		
College/Associate Degree	70	62	19	4		
Bachelor's Degree	76	79	17	8		
Graduate or Above	13	13	4	0		

The results indicate a significant correlation between gender and the degree of awareness of the Heimlich maneuver (p = 0.013). Although males had a slight edge in terms of awareness (72.1% vs. 63.1%), females demonstrated greater proficiency in understanding its application and purpose. Age was also a significant factor influencing awareness (p = 0.005), with the middle-aged group, particularly those aged 31-45 years, showing superior knowledge. This group exhibited higher accuracy and familiarity with the maneuver’s use and operational principles. In contrast, individuals aged 60 and older had limited knowledge, with only a few able to correctly describe key details. Occupation played a notable role in shaping awareness (p = 0.014), with healthcare workers and private employees showing significantly higher levels of knowledge, especially healthcare workers who had a deeper understanding of operational details. Teachers also demonstrated a relatively higher level of awareness, suggesting that professionals in the education sector are more attuned to health-related knowledge. However, the "other" occupational group displayed considerable variability in their level of awareness, likely influenced by the diversity of professions and access to relevant information. Educational background was positively correlated with awareness (p = 0.037). Respondents with higher education levels, particularly those with bachelor’s degrees or above, demonstrated a significantly better understanding of both the operation and principles of the Heimlich maneuver.

Training experience and practical application

Only 27.6% (136 individuals) of the participants reported having received training in the Heimlich maneuver, with a slightly higher training rate among males (30.9%) compared to females (26.0%). Occupational background was significantly related to whether respondents had received training (p < 0.05), with healthcare workers (58.4%) being more likely to have undergone training compared to other professions. Among those who had received training, 34.6% (47 individuals) reported having applied the maneuver in real-life situations, such as emergencies during family meals or in public settings. These findings indicate a significant association between training experience and practical application, although the overall training coverage remains low.

Correlation between awareness and behavior

Awareness was strongly correlated with training (χ² = 24.003, p<0.01), with 45.8% of aware respondents having received training compared to 9.1% of unaware respondents. A significant association was also found between awareness and application (χ² = 7.868, p = 0.049), with 21.9% of respondents who could describe the maneuver’s operational details reporting real-life application, compared to 0% of unaware respondents.

Attitudes and suggestions for promotion

**Figure 1:**
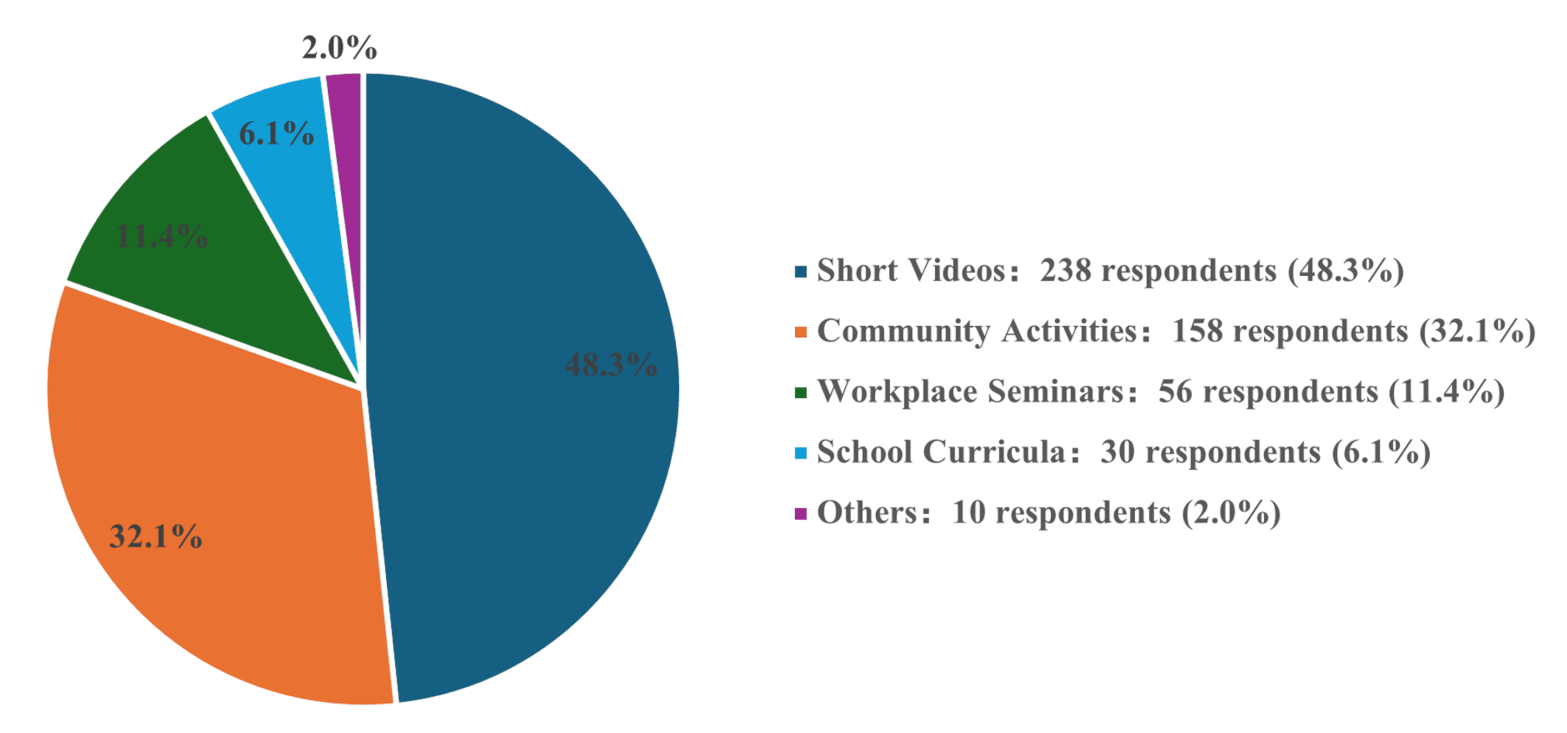
Suggestions for Heimlich maneuver training promotion (N=492) Pie charts show respondent preferences. Statistical analysis used descriptive frequencies.

A total of 81.9% (403) of respondents expressed willingness to learn the Heimlich maneuver, with males showing greater interest (87.3% vs. 79.2% for females; χ² = 8.892, p = 0.031, V = 0.13). Primary motivations included protecting family (62.5%) and assisting others (28.3%). Figure 1 summarizes the preferred learning methods, with short videos (48.3%) and community activities (32.1%) most favored, followed by workplace seminars (12.4%) and school curricula (7.2%).

## Discussion

Airway obstruction caused by foreign bodies has become a significant global health issue, leading to unintentional injuries and fatalities, particularly among children and the elderly. Due to delayed reactions and a lack of first aid knowledge, these vulnerable groups often face fatal outcomes when confronted with airway obstruction [1,4,9]. The widespread adoption and correct application of the Heimlich Maneuver, as a simple and effective first aid technique, are crucial in reducing mortality rates from such accidents [5]. This study aimed to assess the awareness and practical application of the Heimlich maneuver among residents in Shenzhen's communities.

This study revealed that 94.1% of Shenzhen residents were aware of the Heimlich maneuver, yet only 40.9% could perform it, highlighting a significant gap between awareness and practical proficiency. This finding aligns with studies in Saudi Arabia, where high awareness among teachers did not translate to practical skills [6]. Similarly, an Ethiopian study reported limited proficiency among kindergarten staff despite awareness [7]. Shenzhen’s transient population and rapid urbanization may exacerbate this gap, as inconsistent access to training limits skill development. Addressing this requires systematic, hands-on training to bridge the gap between knowledge and application.

Moreover, the study found that public first aid training significantly enhanced residents' emergency response capabilities. Among those who had received training, approximately 34.6% reported applying the Heimlich Maneuver in real-life situations for self-rescue or to assist others. This highlights the vital role of practical training in improving emergency preparedness and reducing injury-related risks [6,7,10]. However, simply disseminating theoretical knowledge is not sufficient to ensure effective emergency responses. Current first aid training programs often lack interactivity, offer limited practical experience, and do not incorporate systematic skill evaluation, all of which impede the mastery and practical application of emergency techniques [7,11,12].

The study also explored the influence of socio-demographic factors on first aid knowledge and skills. Variables such as gender, age, occupation, and education level significantly affected both awareness and training experience. Male participants exhibited slightly higher levels of first aid knowledge, whereas females tended to perform better in operational skills, possibly due to their more active participation in health education initiatives. Middle-aged individuals (31-45 years) demonstrated the highest knowledge levels, likely reflecting greater health awareness and social responsibility within this age group. Additionally, medical professionals and those with higher education levels consistently showed stronger first aid knowledge and practical abilities, underscoring the impact of professional and educational backgrounds. These findings align with studies in Saudi Arabia, where professionally trained teachers exhibited notable advantages in managing choking emergencies [6].

Although the majority of respondents were familiar with the Heimlich maneuver, their practical ability to execute it remained insufficient. Approximately 34.6% of trained individuals reported having used the technique in real-life scenarios, emphasizing the importance of practical application in skill retention and confidence building. Nevertheless, a significant gap persists, which may be attributed to the low frequency of emergencies, individual stress responses, and decision-making challenges during critical moments [13]. Therefore, future first aid education should focus on enhancing real-world responsiveness through simulation exercises and scenario-based drills.

Interestingly, most community residents expressed a high interest in learning the Heimlich Maneuver, with gender playing a significant role in influencing their willingness to learn. Female participants showed a higher inclination to learn, which may be linked to their greater caregiving responsibilities within households. Regarding preferred learning methods, respondents favored short video platforms and community activities, reflecting the current trend of utilizing short videos for health knowledge dissemination [14]. This preference was especially evident among younger populations, who are increasingly relying on short videos for health information. To enhance the reach and engagement of first aid training, public health departments should consider diversifying training methods and tailoring communication channels to different demographic groups. Moreover, the survey revealed that 45.8% of respondents suggested incorporating first aid training into community activities or school curricula, emphasizing the crucial role of communities and schools in spreading first aid knowledge. Integrating first aid education into the curriculum not only strengthens students' and community members' response capabilities but also cultivates their awareness of self-rescue and emergency management [8,15,16]. Respondents also proposed expanding the promotion of first aid knowledge via social media platforms, particularly using easy-to-understand and shareable formats like short videos.

To address the gap between awareness and practical application, and to enhance public proficiency in the Heimlich maneuver, this study proposes the following strategies: (1) Enhancing training accessibility: strengthen the promotion and training of first aid knowledge for different social groups, particularly those with lower educational levels or elderly individuals, through community events, public lectures, and online education, thus reducing learning barriers and improving awareness. (2) Increasing practical opportunities: first aid training should not be confined to theoretical instruction but should incorporate hands-on practice and scenario-based simulations, allowing participants to experience and perform the techniques in realistic situations, thereby improving their ability to respond under pressure. (3) Strengthening emergency response drills: for those already trained, regular refresher courses should be implemented, especially in response to the frequent updates in first aid techniques. Community-based first aid volunteers can be encouraged to further promote training and practical application. (4) Developing high-quality, short-form video tutorials for distribution on popular platforms (e.g., WeChat, Douyin), designed with interactive and scenario-based demonstrations. (5) Strengthening government and social organization collaboration: the government should increase its support for first aid training, providing financial and policy backing, and promote the dissemination of first aid skills through collaborations with medical institutions, schools, and social organizations.

Although this study sheds light on the awareness and application levels of the Heimlich maneuver among community residents in Shenzhen, it does have limitations. First, the study is limited to specific communities in Shenzhen, which may affect the generalizability of the results. Second, while a questionnaire survey was used to collect data, individual subjective assessments of first aid knowledge may introduce biases, potentially affecting the accuracy of the data. Future studies could expand the sample size and adopt more objective evaluation methods (e.g., practical skill assessments) to further explore the effectiveness and improvement strategies of first aid training.

## Conclusions

This study finds that, although Shenzhen residents demonstrate a high level of awareness of the Heimlich Maneuver, there is a notable discrepancy in their practical application abilities. First aid training significantly enhances public competence, particularly among healthcare professionals and individuals with higher educational levels. Promoting first aid skills requires a more systematic and hands-on training approach, integrated with modern communication tools such as short videos and social media, to improve the public's ability to apply first aid skills. Strengthening public health education and promoting the dissemination of first aid knowledge, particularly at the community level, is key to enhancing national emergency response capabilities.

## Appendices

**Table 3:** Questionnaire on awareness and application of the Heimlich maneuver

Question No.	Question Text	Response Options
Demographics		
Q1	Your Gender:	• Male • Female
Q2	Your Age:	• 18-30 years • 31-45 years • 46-60 years • Over 60 years
Q4	Your Occupation:	• Healthcare worker • Teacher • Student • Private enterprise employee • Retired individual • Other
Q5	Your Education Level:	• Primary school or below • Junior high school • High school • College/Associate degree • Bachelor's degree • Graduate degree or above
Awareness & Knowledge		
Q6	Have you heard of the Heimlich maneuver?	• Yes • No
Q7	Are you aware that the Heimlich maneuver can be applied to both children and adults?	• Yes • No
Q8	Do you think it is necessary to learn the Heimlich maneuver?	• Necessary • Not necessary • Other (please specify)
Q9	What is your level of knowledge about the Heimlich maneuver?	• Know how to perform it • Understand its purpose • Have heard the name, but unaware of details • Completely unaware
Q10	What condition is the Heimlich maneuver primarily used to address?	• Cardiac arrest • Airway obstruction • Poisoning • Traumatic bleeding
Q11	What is the principle of the Heimlich maneuver?	• Using pressure generated by abdominal thrusts to force residual air from the lungs to create airflow and expel the foreign object • Restoring heart function through cardiopulmonary resuscitation (CPR) • Expelling the foreign object by inducing vomiting • Unsure
Q12	Is the operational method of the Heimlich maneuver the same for different age groups?	• Yes • No
Q13	In which of the following situations is the Heimlich maneuver NOT suitable?	• Infants (under 1 year old) • Pregnant women • Adults with chest injuries • None of the above
Experience & Application		
Q14	Have you received training in the Heimlich maneuver?	• Yes • No
Q15	If you are familiar with the Heimlich maneuver, have you ever applied it?	• Yes • No
Q16	When an adult experiences airway obstruction, which Heimlich maneuver method(s) should be used? (Multiple choice)	• Standing abdominal thrusts • Supine abdominal thrusts • Back blows • Don’t know
Q17	When an infant experiences airway obstruction, which Heimlich maneuver method(s) should be used? (Multiple choice)	• Back blows • Abdominal thrusts • Chest thrusts • Don’t know
Attitudes & Preferences		
Q18	What is your attitude towards learning the Heimlich maneuver?	• I would like to learn the method, but don’t think it’s very important • Would attend specialized training if given the opportunity • I don’t think I need to learn it • Other (please specify)
Q19	If you encountered someone experiencing airway obstruction due to a foreign object, would you be willing to help them?	• Willing • Very willing • Unsure • Unwilling • Very unwilling
Q20	Regarding learning first aid techniques, what is your biggest concern?	• The learning process is complex and difficult to master • Improper operation may cause additional harm • Lack of practical operation opportunities • Other (please specify)
Q21	Which method do you think is the most effective way to learn the Heimlich maneuver?	• Short videos • Community activities • Workplace seminars • School curricula • Others (please specify)
Open-Ended Feedback		
Q22	Do you have any suggestions or ideas for promoting and popularizing the Heimlich maneuver?	(Free-text response field)
